# Application of Laser-Induced Graphene Flexible Sensor in Monitoring Large Deformation of Reinforced Concrete Structure

**DOI:** 10.3390/s24237444

**Published:** 2024-11-21

**Authors:** Lina Liu, Chenning Cai, Zhenghua Qian, Peng Li, Feng Zhu

**Affiliations:** 1 College of Aerospace Engineering, Nanjing University of Aeronautics and Astronautics, Nanjing 210016, China; liulina@nuaa.edu.cn (L.L.); qianzh@nuaa.edu.cn (Z.Q.); lipeng_mech@nuaa.edu.cn (P.L.); 2 College of Air Transport and Engineering, Nanhang Jincheng College, Nanjing 211156, China; 3 College of Civil Aviation, Nanjing University of Aeronautics and Astronautics, Nanjing 210016, China; 4 Shenzhen Research Institute, Nanjing University of Aeronautics and Astronautics, Shenzhen 518057, China; 5 College of General Aviation and Flight, Nanjing University of Aeronautics and Astronautics, Liyang 213300, China; zhufeng.phd@nuaa.edu.cn

**Keywords:** laser-induced graphene, flexible sensor, transfer process, large deformation monitoring, reinforced concrete structure

## Abstract

When cracks appear in reinforced concrete (RC) structures, the tensile load will be borne by steel bars with high ductility, resulting in a large deformation. Traditional strain sensors have difficulties in achieving good performance for large deformations in concrete structures. In this paper, based on a laser-induced graphene (LIG) technique, a flexible sensor is proposed for monitoring large deformations of concrete structures. Polyimide film is used as the carbon precursor to prepare LIG through laser scanning and then LIG is transferred onto a polydimethylsiloxane (PDMS) substrate to form the flexible sensor. The calibration and performance verification of the flexible sensor are completed through tensile tests. The applicability of the flexible sensor in monitoring large deformations of concrete is verified through beam bending experiments. The fatigue resistance of the flexible sensor is verified through fatigue tests on a full-scale beam. The experimental results showed that the flexible sensor has the advantages of low cost, simple preparation, and stable performance, making it suitable for applications in the field of large deformation monitoring of RC structures.

## 1. Introduction

Reinforced concrete (RC) structures are commonly used in modern architecture. Due to the nature of the material and the influence of the external environment, RC structures may undergo large deformations during use, resulting in cracks that can severely impact their safety and stability [1]. Furthermore, after concrete cracks, the extreme ductility of the internal steel reinforcement (exceeding 15%) allows the structure to undergo substantial deformations (with strains of approximately 10%) under extreme conditions [2]. Even within the normal range of deformation control for RC structures (with strains of approximately 5%) [3], these deformations far exceed the testing limits of conventional strain sensors (e.g., resistance strain gauges with 2% [4], optical fiber sensors with 0.3% [5], and bonded strain gauges with 0.5% [6]). With the rapid development of smart sensing materials, flexible sensors based on novel sensing materials have become a research hotspot in various structural monitoring applications due to their excellent conductivity and piezoresistivity [7,8,9,10].

Among these new sensing materials, graphene has gained a lot of attention since its discovery. For example, Beladi-Mousavi et al. [11] applied an electric field to a two-dimensional graphene oxide (GO) aqueous solution in an electrochemical device containing platinum electrodes, resulting in the preparation of ultralight reduced graphene oxide (RGO) with high conductivity and a high surface area. Smita et al. [12] utilized methanol extract from seaweed as an efficient and green bio-reductant to produce highly stable RGO nanosheets with anti-breast cancer cell toxicity. Cheng et al. [13] designed and fabricated a resistive humidity sensor based on RGO materials using micro-electromechanical system processes such as deposition, photolithography, sputtering, and stripping. The humidity-sensitive material was prepared by uniformly dispersing a certain proportion of RGO powder and dispersant into an aqueous solution through ultrasonic dispersion, demonstrating excellent stability and repeatability through testing. Dong et al. [14] prepared RGO using Hummer’s method and subsequently loaded carbon nanotubes (CNTs) onto the surface of RGO through electrostatic assembly. This was then introduced into a thermoplastic polyurethane (TPU) matrix to create a conductive nanocomposite material, which was further developed into a high-performance flexible piezoresistive sensor for monitoring human physiological activities. David et al. [15] fabricated a multi-sensor array using hole-matrixed carbonylated (C-ny) graphene films as sensing elements, breaking through the limitations of current semiconductor technologies and advanced e-nose technologies. This enabled the selective discrimination of the studied alcohols and demonstrated the long-term performance of the fabricated chip.

Currently, in the material preparation for graphene-based flexible sensors, the traditional reduction–oxidation method is hindered by a myriad of issues, including a complex manufacturing process, chemical residues, the necessity of masking, low yield, and high costs, all of which limit its widespread application and development. Moreover, the dispersion of RGO within polymer matrices may lead to instability in the conductive properties of sensors, thereby compromising their sensitivity and reliability [16]. In 2014, American researchers introduced laser-induced graphene (LIG) technology, which integrates the fabrication and patterning of three-dimensional graphene into a single process. This technology boasts advantages such as environmental friendliness, the elimination of the need for masking, high efficiency, and low cost [17,18,19], making it a promising new direction for the development of flexible and wearable sensors. For example, Jeong et al. [20] generated LIG on the surface of polyimide (PI) film through laser irradiation and then transferred it to a PDMS surface for encapsulation, resulting in a flexible and highly sensitive strain sensor with excellent response capabilities. The sensitivity of this sensor can reach a range of 1 to 160, and its stretchability can reach approximately 30% depending on the degree of the carbonization of the LIG pattern. The feasibility of its application in human motion detection within the industrial context has been investigated. Zhu et al. [21] directly etched PDMS with a laser to create a flexible pressure sensor that can monitor wrist pulses with good stability. The sensor exhibits ultrahigh sensitivity (∼480 kPa^−1^) while maintaining a fast response/relaxation time (2 μs/3 μs) and excellent cycle stability (>4000 repetitive cycles).

Currently, flexible sensors prepared based on the LIG method have been widely applied in various scenarios involving minute deformations, such as personal intelligent medical monitoring, motion tracking systems, and human–machine interaction [22]. However, in the broader context of engineering applications, particularly in the monitoring of fine cracks in reinforced concrete structures, the exploration and application of graphene-based flexible sensors are still in their infancy and require further research and expansion into new application scenarios [23,24].

In this study, a flexible sensor was fabricated by transferring LIG onto a PDMS substrate. The calibration and performance verification of the flexible sensor were completed through tensile tests, and it was subsequently applied to monitor concrete deformation. The correspondence between the resistance variation in the flexible sensor and concrete deformation was verified. Furthermore, the fatigue resistance of the flexible sensor was validated through fatigue experiments on full-scale beams, thereby further exploring the monitoring performance and application effects of the flexible sensor for large deformations in reinforced concrete structures.

## 2. Preparation and Characterization of LIG Material

**Figure 1:**
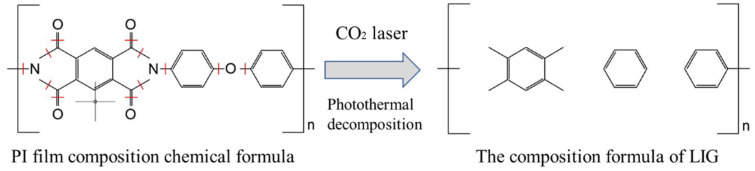
Schematic diagram of LIG molding.

LIG is an emerging technology that utilizes laser irradiation to convert graphite oxide on plastic substrates into graphene [20]. LIG involves scanning various carbon precursors (such as PI films, wood, fabrics, and paper) with lasers (including CO_2_ lasers, ultraviolet lasers, and nano-lasers), leveraging photothermal effects and chemical reduction to decompose oxygen- and nitrogen-containing groups on the substrate. As depicted in Figure 1, this process leads to the saturation and breaking of C-N, C-O, and C=O bonds, leaving only six-membered carbon rings that crosslink and polymerize, resulting in the synthesis of graphene-like structures with six-membered rings. Simultaneously, carbon atoms rearrange from SP_3_ bonds to SP_2_ bonds, forming graphene [21,22,23]. Due to its low cost, excellent mechanical properties, good thermal stability, and resistance to biochemical corrosion, PI film is one of the most commonly used carbon precursors for LIG.

### 2.1. Preparation Process of LIG Material

**Figure 2:**
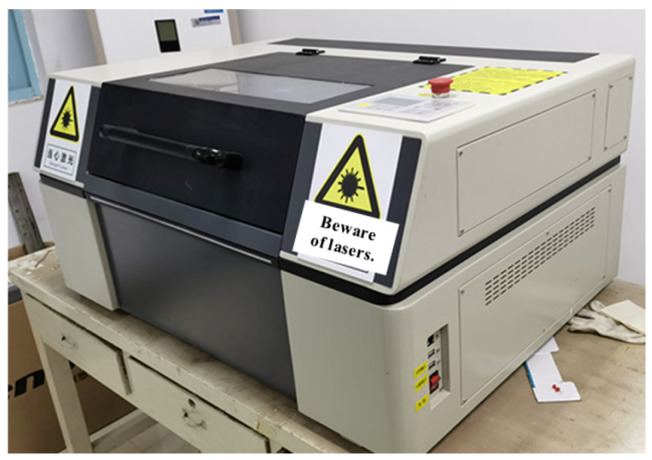
Laser engraving machine.

LIG exhibits high porosity, mechanical robustness, and excellent electrical and thermal conductivity. Its porous structure arises from the generation of gases during the carbonization process on the surface of the source polymer film. The physical and chemical properties of LIG can be tailored by adjusting laser parameters. Generally, increasing laser power enhances the rate of gas release, resulting in a larger number of pores with greater diameters and higher porosity, which subsequently increases the thickness of LIG and enhances its electrical conductivity. However, continuously increasing the laser power beyond the threshold can also lead to the destruction of the LIG structure [24]. In this study, under ambient conditions, a 4060-type laser engraving machine with a rated power of 50 W (as shown in Figure 2) was used to scan PI films (thickness of 0.025 mm) at a scanning speed of 100 mm/s and a scanning density of 400DPI, inducing the formation of graphene patterns. Based on multiple experimental attempts, it was found that LIG exhibits the highest crystallinity, the fewest defects, and the largest graphene domain size at a laser power of 4.8 W. When PI is used as the source polymer, the laser energy required for the initial formation of LIG is 5.5 J/cm^2^.

### 2.2. Characterization of LIG Material

**Figure 3:**
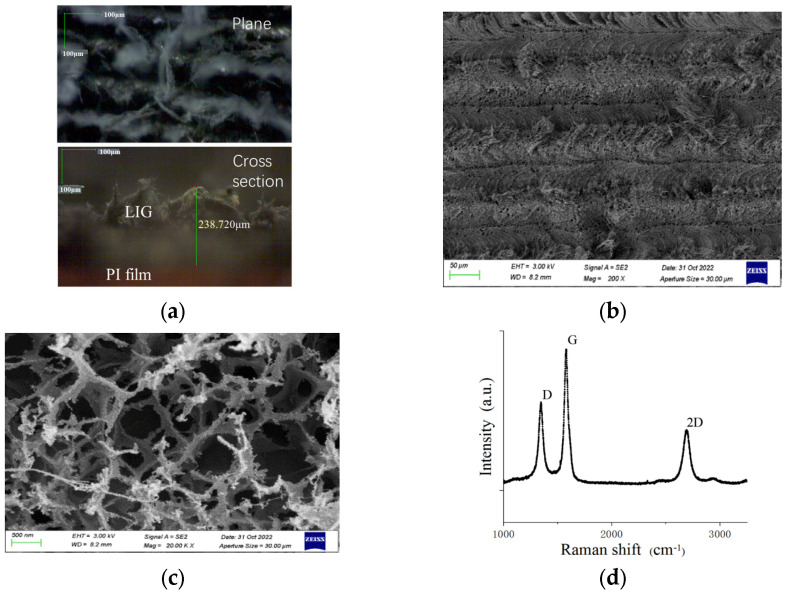
Morphology characteristics and Raman spectra of LIG: (**a**) 540× microscope magnification; (**b**) low-rate SEM morphology characterization; (**c**) high-rate SEM morphology characterization; (**d**) Raman spectral analysis.

The LIG samples prepared on the surface of PI films were characterized for their surface morphology and atomic structure using a digital microscope, a scanning electron microscope (SEM), and Raman spectroscopy, as shown in Figure 3. Figure 3a,b reveal that the surface morphology of LIG exhibits overlapping “spine”-like structures, forming a stable and porous structure with a thickness of approximately 238 μm. Figure 3c shows that the interior of LIG exhibits a porous structure with quasi-circular pores of relatively large diameters, which are caused by the rapid release of gases during the laser scanning process of the PI film. Figure 3d displays three distinct characteristic peaks at 1346 cm^−1^ (D peak), 1578 cm^−1^ (G peak), and 2690 cm^−1^ (2D peak). Among them, the D peak is attributed to defects or curved SP^2^ bonds within the graphene itself, while the G peak arises from the in-plane vibration of SP^2^ carbon atoms [24]. The calculated intensity ratio of the G peak to the D peak (*I_G_/I_D_*) is approximately 1.60, indicating that the prepared LIG material has a relatively low degree of defects, a high structural porosity, and a high degree of graphene crystallinity.

## 3. Preparation and Performance of LIG Flexible Sensor

### 3.1. Preparation of LIG Flexible Sensor

Sylgrade 184 PDMS (Dow Corning, USA) was selected as the transfer medium for the LIG transfer process due to its excellent insulating properties, waterproof quality, strong shear resistance, low toxicity, non-corrosiveness, and minimal shrinkage. Upon curing, it becomes elastic and transparent, allowing for visual inspection and repair, and exhibits stable overall performance [22]. The base component of PDMS is mixed with the curing agent in a weight ratio of 10:1 to form a solution, which is then fully applied to cover the surface of LIG. To ensure that the solution penetrates the nanoscale foam-like pores, an AP-01P oil-free vacuum pump (Shanghai Yalin Science and Technology Co., Ltd.) is used to create a negative pressure environment with a pressure controlled above −0.08 MPa, ensuring complete sealing. Under the negative pressure, numerous bubbles appear on the surface of LIG, indicating that PDMS is gradually infiltrating into the three-dimensional pores. This process continues until all bubbles disappear, indicating the complete penetration of the PDMS solution. The material is then cured at a temperature ranging from 25 °C to 150 °C for 48 h, transforming into a transparent, elastic, and tough material [25].

**Figure 4:**
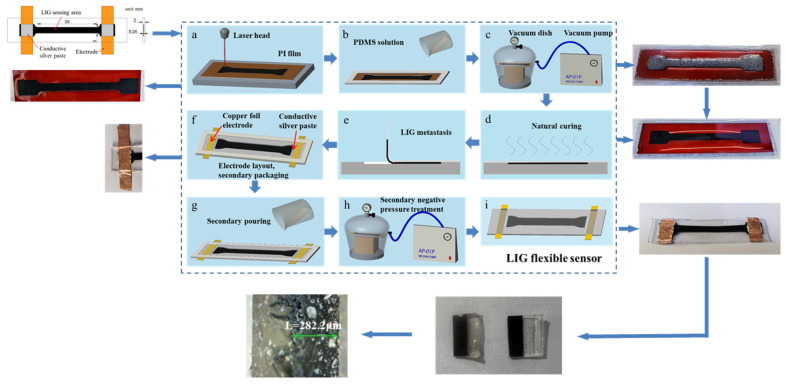
A flowchart for the fabrication of flexible sensors.

Figure 4 illustrates the schematic diagram of the process flow for preparing and transferring LIG onto a PDMS substrate: Firstly, LIG is prepared using a laser engraving machine on a PI film with dimensions of 100 mm × 20 mm, with the theoretical dimensions of the LIG sensing area being 50 mm × 5 mm. Following this, through pouring, vacuum treatment, and curing, the PDMS substrate is peeled off from the PI film surface, resulting in a single-sided elastic LIG/PDMS composite. A conductive silver paste is then used to bond two copper foils to the two ends of the LIG sensing area as electrodes. The PDMS solution is poured again to cover the surface of the elastic composite, followed by repeating the vacuum treatment and curing process. Finally, a flexible LIG sensor is obtained. Cutting the sensor and measuring structure thickness, which is 282.2 μm, slightly increased compared to the original thickness of LIG. It is considered that the reason is that the PDMS solution completely penetrates into LIG, supporting the hollow structure and increasing its thickness. Two samples were prepared in total, one for performance testing and the other for the large deformation monitoring of RC structures.

### 3.2. Performance Test of LIG Flexible Sensor

**Figure 5:**
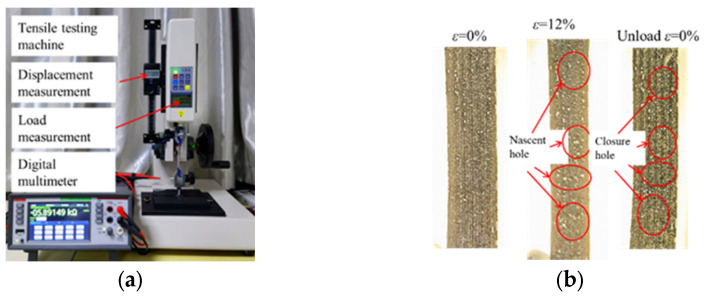
Tensile test of LIG flexible sensor: (**a**) test apparatus; (**b**) LIG-induced layer stretching phenomenon.

To verify the performance of the LIG flexible sensor, a tensile test was conducted using a displacement control method to validate the sensor’s deformation capability and electrical conductivity. The flexible sensor was securely mounted on an HLD-HP1000N tensile testing machine (Aidebao Instrument Co., Ltd.), and the resistance values were monitored using a DMM 6500 digital multi-meter (Keysight Technologies). The initial resistance of the thin film measured using a high-precision digital multi-meter is 9.7 kΩ. Figure 5a depicts the setup for the tensile test.

During the tensile test, observations were made on the microstructure of the LIG sensing layer, as shown in Figure 5b. As the deformation increased, new pores continuously formed and expanded within the LIG sensing layer. Upon unloading, these newly formed pores were able to close and self-heal. However, when the deformation exceeded 12%, some of the original pores merged with the newly formed ones, resulting in a significant increase in the pore area. This led to the formation of pores that could not close or self-heal, causing permanent damage to the structure of the LIG sensing layer. Therefore, the upper limit for testing the LIG flexible sensor was set at 12%.

At the same time, by analyzing the resistance values during the experiment, we can derive the sensitivity coefficient (*K*), which is a crucial indicator for describing strain sensor performance. Typically, this coefficient is calculated based on the ratio of the change in the output electrical signal (either capacitance or resistance) to the applied stress. For resistive strain sensors like the prepared LIG flexible sensor, the sensitivity coefficient (*K*) is determined by the following formula:

(1) $$K=\frac{\Delta R/R_{0}}{\varepsilon }$$

where *R*_0_ represents the initial resistance of the sensor when unstrained, Δ*R* represents the change in resistance after the strain is applied, and *ε* represents the tensile strain.

During the tensile test process, four key points were selected to measure the resistance values and calculate Δ*R*. The results are shown in Table 1.

**Figure 6:**
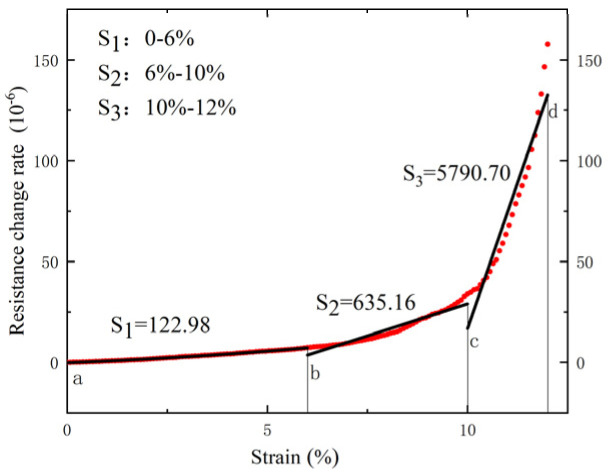
The relation of resistance change rate to strains. (The Δ*R* values for the four key points a, b, c, and d are shown in Table 1.)

As shown in Figure 6. The results indicated a nonlinear relationship between the resistance change rate of the sensor and the deformation. The relationship curve in Figure 6 was fitted using a piecewise linear fitting method into three straight lines with different slopes, where the slopes of these lines correspond to the respective conversion coefficients $S_{n}=K\times 10^{6}$. Through the piecewise linear fitting calculation, the conversion coefficients for the flexible sensor in the low deformation range (0–6%), medium deformation range (6–10%), and high deformation range (10–12%) were determined to be $S_{1}=122.98$, $S_{2}=635.16$ and $S_{3}=5790.70$. Considering this, at the initial stage of the tensile test, the number of newly generated holes of the flexible sensor is small, the expansion of the original holes is small, and the proportional relationship between the resistance change rate and the deformation amount remains stable, showing a good following response.

## 4. Applications of LIG Flexible Sensors

### 4.1. Large Deformation Monitoring of RC Structures

**Figure 7:**
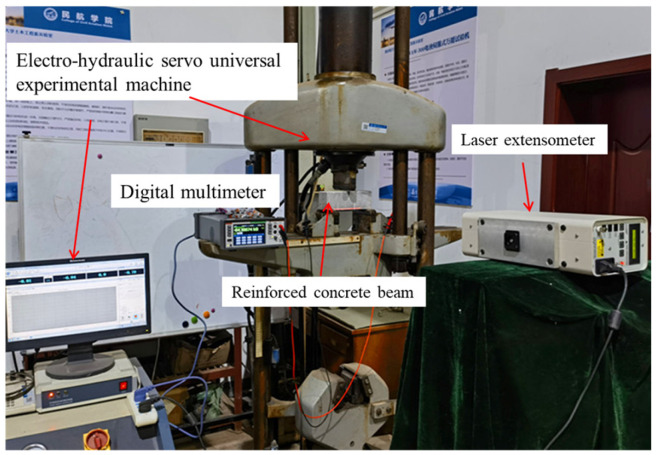
The experimental setup for application performance testing of LIG flexible sensors.

The application performance of the LIG flexible sensor was verified through an RC beam bending test. The test beam was designed in accordance with the “Code for Design of Concrete Structures” (GB50010-2010), with the steel reinforcement configuration and concrete mix ratio designed based on the bending failure mode. The dimensions of the test beam were 70 mm × 70 mm × 210 mm, and a three-point bending load was applied with a support span of 180 mm. The beam was subjected to controlled loads using an electro-hydraulic servo universal testing machine, with the loading mode set to displacement control at a rate of 0.2 mm/min, as shown in Figure 7.

**Figure 8:**
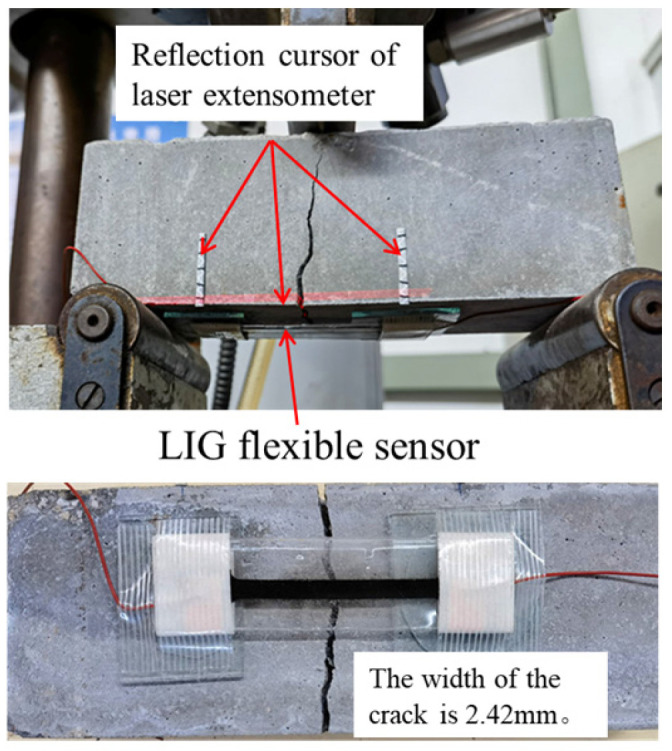
Application of LIG sensor and laser extensometer in RC beam bending test.

To specifically evaluate the LIG flexible sensor’s ability to monitor large deformation areas after cracking in RC, the test beam was first preloaded. Loading was stopped when initial micro-cracks appeared in the tension zone of the beam’s bottom surface. The LIG flexible sensor was then arranged in the area where the initial cracks occurred, with the ends of the sensor adhered and fixed to the concrete surface using hot melt glue, while the central LIG sensing area was left unbonded. The effective gauge length for sensor testing was 50 mm. For comparative evaluation, a reflective target of a laser extensometer was attached to the same monitoring area as the LIG flexible sensor, and the laser extensometer was used to synchronously measure the deformation in this area, as shown in Figure 8.

**Figure 9:**
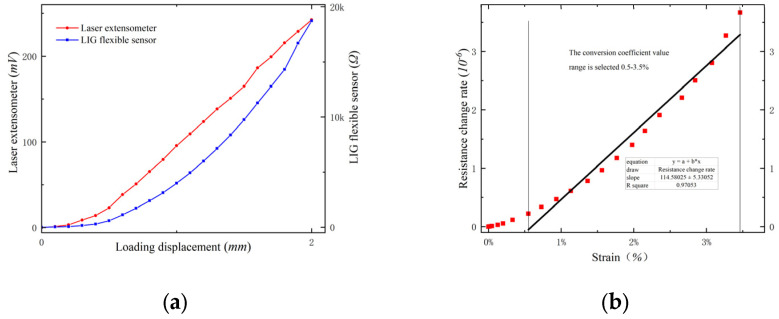
Three-point bending test of RC beam: (**a**) resistance value/output voltage-displacement relationship; (**b**) relation curve between resistance change rate and strain of LIG flexible sensor.

During the beam bending test, a digital multi-meter performed a synchronous test every 30 s. When the loading displacement reached 2.00 mm, the tensile steel bars in the bottom area of the beam yielded; the width of the structural crack was 2.42 mm, indicating a failure phenomenon due to inadequate reinforcement in the normal section of the RC beam; and the test was concluded. Throughout the entire process of the beam bending test, both the LIG flexible sensor and the laser extensometer functioned normally. The relationship curves between the output voltage value of the laser extensometer, the output resistance value of the LIG flexible sensor, and the loading displacement are shown in Figure 9a. The test results indicate that as the displacement loading gradually increased, the development trends of the output voltage value of the laser extensometer and the resistance value of the LIG flexible sensor were basically consistent, and the test results matched well.

By converting the output voltage of the laser extensometer into deformation (%), the maximum deformation was found to be 3.46% [14]. The output resistance of the LIG flexible sensor was converted into a resistance change rate, and a graph of the relationship between the resistance change rate and the corresponding deformation was plotted, as shown in Figure 9b. Analyzing the curve’s conversion coefficient, since the maximum deformation of 3.46% did not exceed the small deformation monitoring range of the LIG flexible sensor, and the initial loading period with imprecise test response of the flexible sensor (deformation less than 0.5%) was excluded from the monitoring range, the resistance change rate and deformation exhibited a linear response. The conversion coefficient was 114.58, with a linearity of 0.9705, which is roughly the same as the value, $S_{1}=122.98$, obtained from the performance verification test. This demonstrates that the LIG sensor can achieve relatively accurate tracking in the monitoring of large deformations in RC structures.

### 4.2. Anti-Fatigue Performance of Flexible Sensors

**Figure 10:**
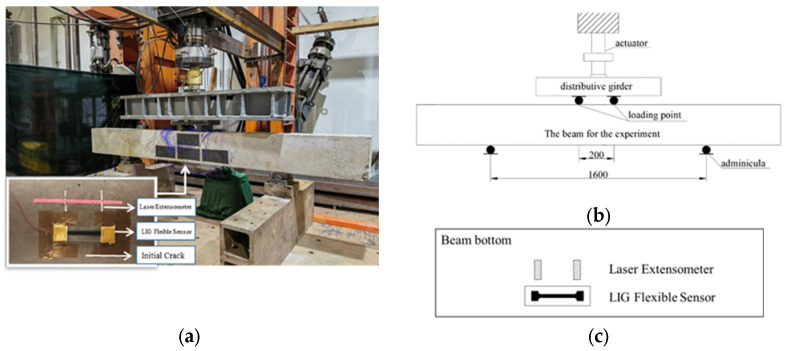
Fatigue test device: (**a**) loading device; (**b**) schematic diagram of loading (unit: mm); (**c**) schematic diagram of sensor arrangement on the bottom of beam.

To further evaluate the durability of the flexible sensor, a fatigue test on a full-scale RC beam was conducted in the laboratory, simulating the entire process from the intact state to the failure of the structure. A multi-channel coordinated loading system was used to cyclically load the RC beam with dimensions of 200 mm × 300 mm × 2700 mm. The loading waveform was sinusoidal, with a loading frequency of 1.0 Hz. A four-point symmetric loading method was adopted, with a spacing of 200 mm between the distribution beams and a support span of 1600 mm. The loading schematic is shown in Figure 10.

**Figure 11:**
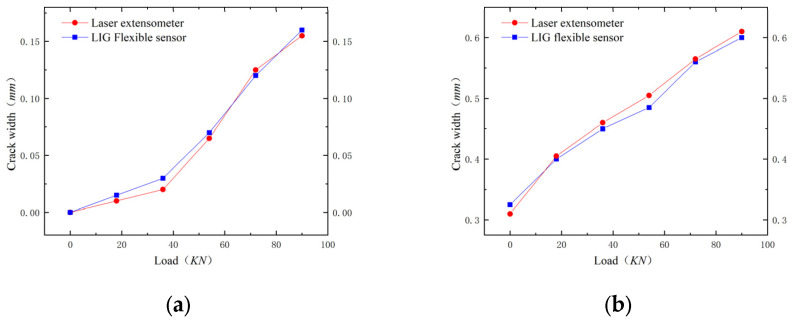
Comparison of test results between LIG flexible sensor and laser extensometer: (**a**) the initial stages of the LIG; (**b**) after 200,000 fatigue loads.

Throughout the fatigue test, after the initial crack appeared, LIG flexible sensors and laser extensometers were arranged at the bottom of the experimental beam, as shown in Figure 10a, and the LIG flexible sensor completed the test synchronously with the experimental beam. To assess whether repeated loading affects the durability of the flexible sensor, we compared the static load test results of the initial stages of the LIG and after 200,000 fatigue loads. The static load test was divided into five levels (18 kN, 36 kN, 54 kN, 72 kN, and 90 kN), followed by unloading to zero load. As shown in Figure 11, the measurement results from the LIG sensor were generally consistent with those from the laser extensometer, exhibiting a good reversible resistance response, stable monitoring performance, and excellent fatigue resistance. This indicates that the sensor is suitable for the long-term deformation monitoring of RC structures.

## 5. Conclusions

In this paper, PI was used as the carbon precursor to prepare LIG through CO_2_ laser scanning. The optimal preparation process parameters were determined through radiation energy analysis and performance characterization. A transfer process was developed to transfer LIG onto a PDMS substrate to create an LIG flexible sensor suitable for large deformation monitoring. The calibration and performance verification of the flexible sensor were completed using tensile tests. The conclusions are as follows:

(1) The preparation method of the LIG flexible sensor is simple and cost-effective. Protected by the PDMS substrate, the sensor is not easily damaged and is suitable for repeated use.
(2) The LIG flexible sensor has a strain measurement range of 12%, which demonstrates its significant effectiveness in monitoring structural cracks and damage.
(3) Through fatigue experiments conducted on full-scale beams, it is further verified that the LIG sensor exhibits an excellent reversible resistance response. After 200,000 cycles of fatigue loading, it still maintains good testing performance, indicating its applicability in the long-term monitoring of large deformations in reinforced concrete beams.

Overall, the LIG flexible sensor can meet the needs of large deformation monitoring in structures. It boasts low preparation costs, a simple testing principle, and stable performance. It is not easily damaged during use and possesses good fatigue resistance, making it suitable for the long-term monitoring of large deformations of concrete structures.

## Figures and Tables

**Table 1:** Relationship between resistance change and strain.

	a	b	c	d
Strain (%)	0	6	10	12
ΔR (Ω)	0	0.07	0.3	1.5

## Data Availability

Data are contained within the article.
